# Plasma Exchange as a Viable Therapeutic Option in a Patient With Alcohol-Related Acute-on-Chronic Liver Failure: A Case Report

**DOI:** 10.7759/cureus.73468

**Published:** 2024-11-11

**Authors:** Aniruddha S Jog, Vikram Jadhav, Vinayak Sawardekar

**Affiliations:** 1 General Medicine, Grant Government Medical College, Mumbai, IND

**Keywords:** acute-on-chronic liver failure (aclf), alcohol-related liver disease, he: hepatic encephalopathy, hrs-aki, severe alcoholic hepatitis, therapeutic plasma exchange (tpe)

## Abstract

Acute-on-chronic liver failure (ACLF) is a severe form of chronic liver disease associated with multi-system organ dysfunction and high short-term mortality. High-volume plasma exchange (PLEX) is one of the therapeutic measures that improves prognosis. We present the case of a 32-year-old man with alcohol-related liver disease who was admitted with acute decompensation, including coagulopathy, hepatic encephalopathy, and acute kidney injury precipitated by alcoholic hepatitis. Despite standard medical treatment, the inflammatory state did not improve. High-volume PLEX was initiated, and five cycles were completed following a multidisciplinary discussion. This intervention improved the clinical and biochemical status of the patient. Pathologically, ACLF is characterized by systemic inflammation, with high levels of cytokines released into the circulation leading to organ failure. In resource-limited settings where liver transplantation is not available, PLEX is emerging as a promising modality, offering biochemical improvement and enhanced survival. PLEX works by eliminating accumulated toxins in ACLF, aiding the recovery of the failing liver and creating a suitable environment for liver regeneration.

## Introduction

Acute-on-chronic liver failure (ACLF) is a severe form of cirrhosis characterized by acute decompensation. It is associated with multi-system organ dysfunction and high short-term mortality [1]. In a multicenter study from India, alcohol was identified as the most common etiology of ACLF, with a 42% mortality rate [2]. Therapeutic measures for patients with alcoholic hepatitis of varying severity, in addition to alcohol abstinence and optimal nutrition, include corticosteroids, pentoxifylline, anti-tumor necrosis factor (anti-TNF) agents, and extracorporeal liver support [3]. Other interventions shown to improve prognosis, according to studies, include plasma exchange (PLEX) and hemodialysis [4,5]. High-volume PLEX has been found to improve transplant-free survival in patients with acute liver failure (ALF) while also enhancing clinical and biochemical variables [6]. We present a case of alcohol-related ACLF (A-ACLF) treated with high-volume PLEX, where liver transplantation was not an option. This case was previously presented as an e-poster at the 2024 BAPIO National Annual Conference on September 20, 2024.

## Case presentation

A previously well 32-year-old male, with no known comorbidities, presented with abdominal distension, yellowish discoloration of the sclera, and bipedal edema for one month. He did not report any chest pain, shortness of breath, reduced urine output, fever with chills, palpitations, loose stools, or vomiting. He had been consuming approximately two-quarters of whiskey daily for the past five years. The patient reported no other addictions.

On examination, his vital parameters were within normal limits. He exhibited pallor, icterus, bipedal edema, and ascites. Blood investigations revealed anemia, thrombocytopenia, elevated bilirubin (total bilirubin = 23 mg/dL), and deranged coagulation parameters (international normalized ratio = 2.3). Ascitic fluid workup indicated a portal hypertensive cause of ascites, with no evidence of spontaneous bacterial peritonitis. Ascitic fluid serum-ascites albumin gradient (SAAG) was 1.4 g/dL, and polymorphs in the ascitic fluid were 10/mm^3^. Noninvasive liver screening, including tests for HIV, hepatitis B, and hepatitis C, was negative. On the tropical fever screen, IgM antibodies to leptospira antigen were detected. However, considering the clinical-serologic disparity, a polymerase chain reaction (PCR) test for leptospira DNA was performed and found to be negative. The autoimmune noninvasive liver screen was also negative. Abdominal ultrasound showed altered liver echotexture with hepatosplenomegaly and ascites.

Initially, he was managed with the best medical care available: intravenous (IV) thiamine, furosemide, vitamin K, oral spironolactone, oral nonselective beta-blockers (NSBB), laxatives, and optimal nutritional therapy. Therapeutic large-volume paracentesis was performed with albumin infusion.

Subsequently, on day 8 of the illness, the patient developed acute kidney injury (AKI). Diuretics and beta-blockers were withheld for 48 hours, but renal parameters did not improve. Suspecting hepatorenal syndrome-AKI (HRS-AKI), IV terlipressin and 20% human albumin solution were initiated. During this time, he also developed symptoms and signs of hepatic encephalopathy (HE) (West Haven grade 3). Given the multiorgan involvement, a hepatology consultation was sought. Due to the unavailability of a liver transplant, combined with renal dysfunction, high-volume PLEX was recommended following a multidisciplinary discussion with hepatology and renal medicine specialists.

The patient received five cycles of high-volume PLEX, utilizing fresh frozen plasma and employing membrane filtration for PLEX. Blood investigations pre- and post-cycle (Table 1) demonstrated significant improvement. Clinically, he showed reduced ascites and peripheral edema, resolution of AKI, and a marked decrease in serum bilirubin levels.

**Table 1:** Trends of FBC, liver function test, and renal function test during the course of illness Hb, hemoglobin; WBC, white blood cells; PLT, platelets; AST, aspartate transaminase; ALT, alanine transaminase; FBC, full blood count.

Day	Hb (g/dL)	WBC (per µL)	PLT (per µL)	AST (IU/L)	ALT (IU/L)	Total Bilirubin (mg/dL)	Urea (mg/dL)/Creatinine (mg/dL)	PT (seconds)/INR
On admission	12.3	13,700	151,000	129	61	23.9	20/0.7	31/2.3
Day 12	9.5	12,500	99,000	124	45	46	41/1.2	27.4/1.87
Day 14	10	15,400	68,000	135	45	45.5	60/1.9	
Post-PLEX Session 1	7.4	7500	63,000	59	24	28.9	40/0.6	20.1/1.36
Post-PLEX Session 2	6.9	10,300	35,000	83	28	22.6	41/0.7	20/1.3
Post-PLEX session 3	7.1	3400	34,000	68	38	20.4	31/0.7	23.1/1.57
Post-PLEX session 4	9.1	6700	44,000	54	28	16.7	32/0.7	
Post-PLEX session 5	8.1	6600	46,000	21	50	12.7	33/0.6	
Post-PLEX Day 7	9.2	5900	110,000	69	28	14.2	15/0.7	18/1.2
1 week after discharge				45	15	17.9	30/0.8	
1 month after discharge				58	32	10.9	28/0.9	
2 months after discharge				40	38	5.1		

He was then continued on best medical management and optimal nutrition. Diuretics for ascites were reinitiated at the lowest effective doses, and his abdominal girth, fluid status, along with a strict record of intake and output (to maintain a slight negative fluid balance), renal parameters, and electrolyte panels were closely monitored. Peripheral edema and ascites reduced significantly; however, his serum bilirubin levels persisted within the range of 12 to 16 mg/dL. Despite this, the patient was clinically close to his functional baseline and showing improvement. Consequently, he was discharged with a follow-up in the hepatology clinic for evaluation for a liver transplant. On follow-up, his liver parameters showed improvement (refer to Table 1).

## Discussion

ACLF is a relatively newly defined concept. From a pathophysiological perspective, the syndrome represents an interplay between systemic inflammation and an altered host response to injury. Clinically, it can be described using the Predisposition, Injury, Response, and Organ Failure (PIRO) concept [7]. Acute decompensation results from extremely high levels of plasma cytokines and oxidized albumin, with ACLF being associated with a further rise in these markers [8]. PLEX helps eliminate accumulated toxins in ACLF patients and facilitates recovery of the failing liver by creating a milieu conducive to liver regeneration [9]. According to one study, 30- and 90-day mortality related to liver failure was averted by PLEX. This modality was also superior to standard medical treatment, lowering the risk of multi-organ involvement and improving survival [10]. In another study, patients with A-ACLF who received PLEX demonstrated a higher survival rate compared to those on standard medical therapy. Additionally, 60% of patients in the PLEX group with abnormal creatinine levels showed normalization of creatinine, which remained in the normal range during follow-up [11].

In our case, we had a patient presenting with the first episode of alcohol-related liver disease and decompensation. Initially, best medical management was initiated, including diuretics, NSBB, and aggressive nutritional therapy. Despite this, the patient developed multiorgan dysfunction, including acute kidney injury (AKI) and hepatic encephalopathy. Steroids were not considered due to the patient's A-ACLF, as administering steroids in patients with higher A-ACLF grades carries a greater risk of infection and diminished responsiveness [1].

Due to resource constraints, a liver transplant was not an option. Given the patient's A-ACLF with large-volume ascites, a multidisciplinary team recommended high-volume PLEX. A total of five cycles of PLEX were performed. After each successive cycle, there was significant improvement in biochemical parameters, including reductions in bilirubin and improvements in coagulation profile. AKI resolved and did not recur after completing the five cycles of PLEX. The patient’s full blood count, which showed bi-cytopenia, also improved. By the time of discharge, the patient was reinitiated on low-dose diuretics and continued aggressive nutritional therapy with a high-protein diet. The patient was followed up as an outpatient for two months post-discharge, during which liver function tests showed significant improvement in bilirubin levels.

## Conclusions

This case highlights the importance of understanding the pathophysiology of a disease process when faced with a challenging clinical scenario. It also encourages consideration of possible approaches to address complex pathophysiology using available resources. In our case, a liver transplant was not a feasible option due to resource constraints. However, a deep understanding of the pathophysiology of ACLF and consideration of other available treatment modalities, such as PLEX, enabled us to manage this complex clinical situation effectively. PLEX may thus be considered a viable option for patients presenting with A-ACLF in resource-constrained settings, with specialist guidance.

## References

[1] Moreau R, Tonon M, Krag A (2023). EASL clinical practice guidelines on acute-on-chronic liver failure. J Hepatol.

[2] Saraswat V, Singh SP, Duseja A (2016). Acute-on-chronic liver failure in India: the Indian National Association for Study of the Liver Consortium experience. J Gastroenterol Hepatol.

[3] Horie Y (2012). Granulocytapheresis and plasma exchange for severe alcoholic hepatitis. J Gastroenterol Hepatol.

[4] Horie Y, Yamagishi Y, Ebinuma H, Hibi T (2011). Therapeutic strategies for severe alcoholic hepatitis. Clin Res Hepatol Gastroenterol.

[5] Horie Y, Ishii H, Hibi T (2005). Severe alcoholic hepatitis in Japan: prognosis and therapy. Alcohol Clin Exp Res.

[6] Karvellas CJ, Stravitz RT (2016). High volume plasma exchange in acute liver failure: dampening the inflammatory cascade?. J Hepatol.

[7] Jalan R, Stadlbauer V, Sen S, Cheshire L, Chang YM, Mookerjee RP (2012). Role of predisposition, injury, response and organ failure in the prognosis of patients with acute-on-chronic liver failure: a prospective cohort study. Crit Care.

[8] Clària J, Stauber RE, Coenraad MJ (2016). Systemic inflammation in decompensated cirrhosis: characterization and role in acute-on-chronic liver failure. Hepatology.

[9] Maiwall R, Moreau R (2016). Plasma exchange for acute on chronic liver failure: is there a light at the end of the tunnel?. Hepatol Int.

[10] Maiwall R, Bajpai M, Choudhury AK (2021). Therapeutic plasma-exchange improves systemic inflammation and survival in acute-on-chronic liver failure: a propensity-score matched study from AARC. Liver Int.

[11] Kumar SE, Goel A, Zachariah U (2022). Low volume plasma exchange and low dose steroid improve survival in patients with alcohol-related acute on chronic liver failure and severe alcoholic hepatitis - preliminary experience. J Clin Exp Hepatol.

